# A Commission to Inquire into the Condition of the Colored Population

**Published:** 1863-10

**Authors:** 


### A COMMISSION TO INQUIRE INTO THE CONDITION OF THE COLORED POPULATION.

A Commission has been appointed by the Government to inquire into the condition of the colored population emancipated by the President's Proclamation and by the Act of Congress, and to report what measures are necessary to place them in a condition of self-support and self-defence, with the least disturbance to the great industrial interests of the country.

This Commission are seeking to ascertain the vital statistics concerning the African race, and the mulattoes, as well in the Northern and Middle as in the Southern States.

It is very important that this should be done, but unfortunately the data do not exist.

It is not known, from any wide circle of observation, whether the mulattoes are as fertile as blacks and whites; whether they are long lived; nor even whether their breed can exist permanently; that is, whether its hybridity will prevent its persistence. Then there are questions about the adaptation of the cross-beeed to the northern parts of the temperate zone; questions about the effects of amalgamation upon the white race, and the like.

The Commission have sent out circulars to many medical men, but of course will not reach all who might, if called upon, give valuable aid. We therefore print the series of questions put forth by Dr. Howe in behalf of the Commission, and invite the attention of our readers to it.

Those who are disposed to answer the queries, or to favor the Commission with their views upon the general subject committed to it, are invited to address Dr. Samuel G Howe,
143 Second Ave., cor. East 9th St.,
New York.

QUESTIONS.

1—What is the number of the colored population of your town?

2—About how many pure blacks?

3—Abut how many mulattoes?

4—Does the colored population, if not recruited by immigration, increase or decrease?

5—Do mulattoes seem to you to have as much vital force to resist disease and destructive agencies as pure blacks, and as whites? and do they usually live as long?

6—To what diseases do mulattoes seem peculiarly liable?

7—Do mulatto families usually have as many children as white families?

8—Can you give instances, within your own knowledge, of the number of children in one family born of, and reared to maturity by, mulatto parents?

9—Are the colored people generally industrious and self-supporting, or not?

10—How is it in the second generation with regard to the number and health of offspring?

11—Through how many generations has any family of mulattoes been known to persist?

12—Do the mulalttoes seek public charity in greater or less proportion than whites?

13—Do you consider them, upon the whole, as valuable members of the community, or not?

